# Emotional Intelligence and Personality Traits of University Students in Dentistry, Medicine and Pharmacy Degrees

**DOI:** 10.3390/ejihpe14060116

**Published:** 2024-06-17

**Authors:** Cristina Gómez-Polo, Javier Montero, María Portillo Muñoz, Maria Lobato Carreño, Beatriz Pardal-Peláez, Álvaro Zubizarreta-Macho, Ana María Martín Casado

**Affiliations:** 1 Department of Surgery, Faculty of Medicine and Dentistry, University of Salamanca, 37008 Salamanca, Spain; crisgodent@usal.es (C.G.-P.); javimont@usal.es (J.M.); mlobato@usal.es (M.L.C.); bpardal@usal.es (B.P.-P.); alvaro.zubizarreta@usal.es (Á.Z.-M.); 2 Department of Statistics, Faculty of Medicine and Dentistry, University of Salamanca, 37008 Salamanca, Spain; ammc@usal.es

**Keywords:** emotional intelligence, personality traits, university students, dentistry, medicine

## Abstract

Background: This study aimed to characterize dentistry, medicine and pharmacy students in terms of emotional intelligence (EI) and personality traits (PTs). It also sought to identify whether differences existed according to gender and degree program and the relationship between them. Methods: Students enrolled in dentistry (115), medicine (85) and pharmacy (57) degree programs participated voluntarily in the research, including 59 men and 198 women. The following questionnaires were used: (1) the Trait Meta-Mood Scale (TMMS-24) to evaluate EI; (2) the NEO Five-Factor Inventory (NEO-FFI) to assess PT. The Qualtrics XM platform was used for data collection. Results: There were no statistically significant differences between three components of EI, either according to gender or degree program. The only difference in PTs was found in neuroticism, where women scored higher than men. There were statistically significant differences between students on different degree programs in openness to experience and responsibility. The five PTs correlated significantly with the three components of EI, except responsibility and emotional attention. The strongest associations were found between neuroticism and emotional repair (−0.439). Conclusions: High percentages of the student population were observed to have weaknesses in emotional clarity and emotional repair. Neuroticism is a personality trait that seems to occur more frequently in women.

## 1. Introduction

University-level education has changed significantly in recent years, as students have moved to the center of the learning process. Nevertheless, the pedagogical literature for higher education is not very extensive, with most studies focusing on primary and secondary education [1]. To ensure that health-science students become effective healthcare professionals, they need to leave university equipped not only with complex theoretical knowledge, technical skills and surgical expertise, but also with the ability to effectively communicate and empathize with patients and family members [2,3,4,5,6,7,8]. It is therefore necessary to design an educational model that trains students to be emotionally competent and aware of their strengths and areas for improvement when it comes to their own feelings [9,10]. This is especially significant for undergraduate students in health-science disciplines, who have been reported to suffer from high levels of stress, with pressures relating to the theoretical workload, preclinical and clinical practicals, and hospital placements, to name just a few [11,12]. Within this context, engaging with emotional intelligence (EI) is particularly important. This concept can be defined as a form of social intelligence, through which people are able to recognize their own emotions and other people’s feelings. This emotional awareness enables them to make appropriate decisions, informing their thoughts and actions [13]. Recent studies have investigated EI from different perspectives. For example, students with high EI scores have been observed to have lower levels of depression, stress and social anxiety; view stressors as less threatening [14,15,16,17,18]; deploy a broader range of coping strategies [14,15]; and have positive interpersonal relationships [15], superior academic performance [4,19,20,21], better interpersonal communication skills [22], a higher level of patient loyalty [3,23], and lower missed or cancelled appointment rates [24,25].

The abilities to empathize with patients, engage in active listening, respond effectively to their needs and demands, and provide information to patients and family members accurately and sensitively are indispensable qualities for health professionals to perform competently, without allowing situations to affect them negatively on a personal level [26]. The level of patient satisfaction forms the basis of several quality measures for healthcare programs [27]. Patient satisfaction is related positively and directly to clinicians’ ability to manage the emotions of patients and their relatives [28,29], from which it follows that levels of patient satisfaction are higher when healthcare professionals score higher in EI.

It has even been claimed that a person’s EI quotient comprises 80% of the factors determining success [30]. On this basis, scholars have inferred that it is not possible to predict significant outcomes of individual lives using traditional cognitive intelligence measures, while EI can often be more useful [31]. This functionalist view of emotions can help with problem solving and facilitate adaptation in a context of constant change [32]. In general, high levels of satisfaction and positive opinions among patients are shaped by both EI [27,29,33,34] and personality traits (PTs) [24,35,36]. Personality traits have even been related to the admission rates and productivity levels of private clinics [24] and have been ascribed the ability to predict professional behavior. Researchers have attempted to determine the role of personality traits in academic and employment settings, suggesting that certain personality types may feel drawn to similar employment contexts that seem compatible with their values and interests [24,37]. Zweig and Webster [38] have argued that these individual personality differences are probably the most important factors shaping learning, performance and behavior, considering that controversial matters concerning thought, intelligence, perception, emotions, learning and motivation are all informed by this issue [39]. Other authors have made the case for conducting obligatory personality tests during the residency stage of medical training, given their observed utility in determining performance [40]. Recent studies have highlighted the importance of optimizing emotional intelligence through training and of analyzing personality traits [41] in students in health-science disciplines, given their exposure to stressful academic and clinical situations. Nevertheless, few studies exist that analyze EI and PT in students enrolled in multiple degree programs [42,43] in the biomedical field, where the interpersonal and social components of work and learning are paramount. Consequently, the present study aims to meet the following objectives:

1. To characterize university students in dentistry, medicine and pharmacy degrees according to their emotional attention, clarity and repair (the components of EI) and analyze whether differences exist related to gender and degree program.
2. To characterize university students in dentistry, medicine and pharmacy degrees according to the five personality traits in the NEO Five-Factor Inventory (NEO-FFI) and analyze whether differences exist related to gender and degree program.
3. To analyze the relationship between the three components of EI and the five personality traits and identify the PTs that are associated with EI.

### Research Questions

1. Are there statistically significant differences in any of the components of emotional intelligence (EI) based on gender or degree program?
2. Are there statistically significant differences in any of the five personality traits based on gender or degree program?
3. Are the components of EI correlated with the personality traits among dentistry, medicine and pharmacy students?

## 2. Materials and Methods

The present study was approved by the Institutional Bioethics Committee (registered approval number 673-2021). The Qualtrics XM online survey platform was used for data collection, which was conducted between January 2023 and September 2023.

All students enrolled in dentistry, medicine and pharmacy degree programs were informed of the study and asked to participate voluntarily. Participants were asked to read the instructions and information about the study objectives before clicking on a link in their institutional email to access the content, which was presented in three sections: (1) age, gender and degree program; (2) the TMMS-24 (Trait Meta-Mood Scale-24) emotional intelligence questionnaire; and (3) the NEO Five-Factor Inventory (NEO-FFI) to collect data on personality traits, designed by Costa and McCrae [44].

### 2.1. Instruments Used

(1) The TMMS-24 (Trait Meta-Mood Scale-24) emotional intelligence questionnaire [45,46] is based on the original model designed by Mayer and Salovey (1990) [45] and may be regarded as the first instrument to measure overall emotional intelligence, as well as the distinct components of the concept. There are 24 items in the questionnaire, with responses on a five-point Likert scale (between 1 = strongly disagree and 5 = strongly agree). TMMS respondents are given scores for three dimensions of EI, as follows: “emotional attention” describes how aware respondents are of their emotions, how well they recognize their feelings and their ability to understand what those emotions mean; “emotional clarity” concerns respondents’ ability to identify and understand their emotions, distinguish between them, understand how they change and incorporate them into their thoughts, and “emotional repair” describes how well respondents regulate and control positive and negative feelings.

The TMMS-24 assessment manual categorizes respondents into three groups for each EI component as follows:

- According to the emotional attention score obtained: group 1, respondents who pay little attention; group 2, respondents who pay adequate attention; and group 3, respondents who pay too much attention.
- According to the emotional clarity score obtained: group 1, respondents who need to improve their clarity; group 2, respondents with adequate clarity; and group 3, respondents with excellent clarity.
- According to the emotional repair score obtained: group 1, respondents who need to improve their repair; group 2, respondents with adequate repair; and group 3, respondents with excellent repair.

(2) The NEO Five-Factor Inventory (NEO-FFI) on personality traits (Costa & McCrae, 1992) [44]. The NEO-FFI was adapted to the Spanish population by Cordero, Pamos and Seisdedos (1999) [47]. This instrument is an abridged version of the NEO Personality Inventory, containing 60 items whose possible responses range from 1 = almost never to 4 = almost always. It evaluates the five personality traits, neuroticism, extraversion, openness to experience, agreeableness, and conscientiousness, as described below.

- Neuroticism (N): The converse of this is emotional balance and stability. Neuroticism is defined by a general tendency to experience negative feelings and psychological suffering (such as fear, melancholy or sadness, shame, anger, a sense of guilt and revulsion).
- Extraversion (E): Extraverted individuals are very sociable, generally enjoy being with people and show a preference for groups and meetings. They are assertive, active and talkative, are often of a cheerful nature, find it easy to enjoy themselves, and are optimistic, enthusiastic and energetic.
- Openness to experience (O): People with this trait tend to view experiences positively and are as interested in the external world as the internal world (adventurous nature). They tend to be intellectually curious, imaginative and have high levels of esthetic sensibility.
- Agreeableness (A): This trait says a lot about how people relate to each other (psychosocial bonds and concern for others), evaluating how individuals approach interpersonal relationships (whether they tend to be kind, sensitive, compassionate, etc.).
- Conscientiousness (C): Individuals with this trait are usually determined to achieve their objectives, organized, disciplined, hard-working, responsible, reliable, tenacious, decisive, punctual, ambitious and goal-oriented in their behavior.

Both the questionnaires used in the present research have a high level of reliability and validity.

### 2.2. Description of the Sample

The sample was made up of dentistry (n = 115), medicine (n = 85) and pharmacy (n = 57) students who completed the TMMS-24 emotional intelligence and NEO-FFI personality traits questionnaires. The total sample of 257 students included 59 men (23.0%) and 198 women (77.0%). Within this sample, there were 86 participants aged between 18 and 20 (33.5%), 131 aged between 21 and 23 (51.0%), 20 aged between 24 and 26 (8.2%) and 19 aged over 26 (7.4%).

### 2.3. Statistical Analyses

Descriptive statistics, such as the mean and standard deviation, were used to analyze the data. Comparisons were performed using the following statistical techniques: the unpaired Student’s *t*-test, the one-way ANOVA with a completely randomized design, and the test for homogeneity of proportions [48]. Post hoc comparisons were made using Duncan’s multiple range test [49]. The association between variables was analyzed by calculating the linear correlation coefficients, and Cohen’s criteria (Cohen, 1988) was then used to evaluate them (0.10: small effect size; 0.30: medium effect size; 0.50: large effect size) [50]. SPSS statistical software (version 28) was used to perform all statistical analyses. The significance level was set at 0.05.

## 3. Results

### 3.1. Description of the EI Components among the University Students in the Sample: Differences According to Gender and Degree Program

Table 1 shows the descriptive statistics for scores in the three EI components (emotional attention, clarity and repair) in the total sample, according to gender and degree program, as well as the results of the statistical comparisons performed.

Gender

There were no statistically significant differences between the mean scores of men and women for any of the three EI components.

Degree program

There were no statistically significant differences between the mean scores of students in dentistry, medicine and pharmacy degrees.

However, despite the absence of statistically significant differences between men and women in the mean scores of any of the three EI components, the comparison of the percentages of men and women in each group established by the TMMS-24 assessment manual for each EI component revealed statistically significant differences in the emotional repair group (χ^2^ = 8.481, *p* = 0.014). More women need to improve their emotional repair abilities than men (56.1% versus 37.3%).

No statistically significant differences were found between the percentages of dentistry, medicine and pharmacy students in each group for any of the EI components.

### 3.2. Description of the Five Traits in the Personality Questionnaire among University Students in Our Study Sample: Differences According to Gender and Degree Program

Table 2 shows the descriptive statistics for scores for the five personality traits (neuroticism, extraversion, openness to experience, agreeableness and conscientiousness) in the total sample, according to gender and degree program, and the results of the statistical comparisons performed.

Gender

There were statistically significant differences between the mean scores of men and women for neuroticism. For the other four personality traits, the differences were not statistically significant.

Degree program

There were no statistically significant differences between the mean scores of dentistry, medicine and pharmacy students for neuroticism, extraversion or agreeableness. However, there were statistically significant differences between these student bodies for openness to experience, where medicine students obtained higher scores than students in the other degrees, at 44.60 versus 40.44 and 41.12. Statistically significant differences were also found for conscientiousness, for which dentistry and medicine students obtained higher scores than pharmacy students: 44.08 and 43.31 versus 40.82.

### 3.3. Analysis of the Relationship between Emotional Intelligence and the Five Personality Traits

To analyze the association between each component of EI and each PT, the Pearson correlation coefficients (r) were calculated between each of the three EI components and each PT (Table 3).

Neuroticism had a positive correlation with emotional attention and a stronger negative correlation with emotional clarity and emotional repair, all of which were significant. This showed that students who are least competent in understanding and regulating emotions generally score higher for neuroticism. A significant, positive association was found between all three EI components and extraversion, openness to experience and agreeableness, respectively. For both extraversion and agreeableness, the strongest of these correlations was with emotional repair, indicating that students who better regulate their emotions tend to be more extraverted and agreeable. Openness to experience correlated most strongly with emotional attention, indicating that students who pay more attention to their emotions are also generally more open to experience. Finally, conscientiousness correlated positively and significantly with emotional clarity and emotional repair, but the association was weak in both cases.

## 4. Discussion

This research aimed to study and correlate the non-cognitive factors of emotional intelligence and personality traits in a higher-education context. This information is vital to determine whether students in the degree programs examined have the qualities that contribute most to academic and professional success.

While the student participants belonged to a single university, this is one of the few studies to provide results on several degree programs in health-science disciplines. The sample sizes for the three degrees analyzed were not identical, but they aligned with those of similar studies [51,52,53,54]. All participants in the sample were of Caucasian race and had similar social backgrounds, although there were more women than men (77.0% versus 33.0%). This reflects the larger female student populations in health-science degrees and the gender balance in healthcare professions.

The first null hypothesis of this study should be partially rejected. While no statistically significant differences were found between the mean overall scores for any of the three components of EI, according to gender or degree program, gender differences were identified for emotional repair when the comparison was made between students grouped by their scores for each component, as indicated in the TMMS-24 assessment manual. According to the results, women needed to improve their emotional- epair skills more than men (56.1% versus 37.3%).

The second null hypothesis should also be partially rejected. Gender differences were only found for neuroticism (women scored higher than men for this personality trait), while the comparison of students by degree program identified differences in openness to experience and conscientiousness.

The third null hypothesis should be rejected, given that significant linear associations were found between each of the EI components and several of the personality traits. The personality trait showing the weakest relationship with EI was conscientiousness, while the strongest association was between neuroticism and EI.

### 4.1. Gender and Degree Programs

None of the three EI components differed significantly between male (n = 59) and female (n = 198) students, as observed by Abe et al. (2018) in a Japanese context [54].

Several studies examining the relationship between ability EI [55,56] and gender have concluded that women generally score higher for self-perceived EI [53,57,58,59,60,61]. However, examining the results in greater detail, men tend to score higher in self-perceived emotional repair [62,63], in line with the present results (56% of women needed to “improve emotional repair” compared to 37% of men). Other authors have also suggested that men and women do not necessarily differ in overall emotional intelligence, but in the components thereof [64,65].

Our study found no statistically significant gender differences in personality traits, with the exception of neuroticism, for which women obtained higher scores than men, supporting Cuartero and Tur’s (2021) results [41]. In contrast, Abe et al. (2018) have found women to score higher for agreeableness as well as neuroticism [54].

Some research has reported, albeit not without controversy, that some personality traits are related to specific occupations, meaning that differences in individual personality traits could affect professional success. For example, Rodríguez and colleagues (2017) found that extraversion correlated significantly and positively with the clinical productivity of dentistry students (n = 92) and negatively with their missed appointment rate [24].

### 4.2. The Relationship between Emotional Intelligence and Personality Traits

Our research has also identified a close relationship between the three components of self-perceived ability EI and the Big Five personality traits. The five personality traits were observed to have positive relationships with all three EI dimensions (except neuroticism, which was associated negatively with emotional clarity and emotional repair), with a small-to-medium effect size (from 0.13 to −0.44). The negative relationships between EI and neuroticism were particularly strong [41], suggesting that EI has a protective role, fostering emotional stability. The generally close associations support EI theory, which locates the emotional traits and abilities that constitute (trait) EI at the foundational level of personality. However, the present research obtained lower Pearson correlation coefficients than studies focused on trait EI have. For example, Sambol et al. (2022) found correlations between the five PTs and trait EI ranging from 0.31 (openness) to −0.56 (neuroticism) [66]. On this point, authors have expressed some concern about the potential overlap between the main personality traits and EI, especially when the trait EI model and self-reporting instruments are used [67,68].

### 4.3. Emotional Intelligence Training and Future Lines of Research

Several studies have shown that interventions can enhance EI [41,54,69,70,71,72,73,74,75,76,77,78], with enormous benefits for children, teenagers and adults.

The large percentage of students in all degree programs with low scores in several components of ability EI suggests that EI training activities could have significant benefits. These activities should focus on emotional clarity (which needs to be improved in 66.1%, 57.6% and 59.6% of dentistry, medicine and pharmacy students, respectively) and emotional repair (which needs to be improved in 57.4%, 49.4% and 43.9% of dentistry, medicine and pharmacy students, and particularly in women, as significant gender differences were found).

Developing experimental longitudinal studies on how training programs can improve EI scores in higher-education settings would be helpful to understand how to help students improve interpersonal skills such as communication, with the goal of improving patient satisfaction [27]. To increase such programs’ success, it would be important to identify how to incorporate them into degree curricula, determine which student populations to target [69], and develop a continuing-professional-development plan that helps university educators meet these new challenges [79].

The educational benefits of this type of EI intervention—including improved teacher–student relationships, emotional education and fewer behavioral issues—have been demonstrated from kindergarten to high-school level [80]. Their value for university education may be assumed, although empirical evidence is still lacking. Likewise, educators who are aware of learners’ emotional states may manage student groups better and choose more effective teaching strategies [81]. Such interventions should not be limited to specific courses but form part of a pedagogical vision that places learners at the center of higher-education goals throughout their university careers. It is vital for today’s students to improve their emotional intelligence to ensure that future clinicians are equipped to understand patients and family members and provide effective, supportive emotional management for them. To optimize the effect of these EI-development activities, personality traits need to be taken into account.

## 5. Conclusions

Promoting activities to improve the EI of students in health science degrees would be beneficial, due to the high percentage of the student population with weaknesses in emotional clarity and emotional repair. Neuroticism is a personality trait that appears to occur more frequently in women. It is vital for today’s students to enhance their emotional intelligence to ensure that future clinicians are equipped to understand patients and family members, providing effective, supportive emotional management for them. To optimize the effect of these EI-development activities, personality traits need to be taken into account. The primary limitation of this research is its purely descriptive focus, precluding its use for predicting behaviors.

It is recommended that university management bodies invest human and economic resources in studying and analyzing these types of psychological constructs to optimize students’ well-being and academic performance.

## Figures and Tables

**Table 1:** Summary of descriptive statistics and statistical comparisons related to the scores for each component of EI, according to gender and degree program. (SD: Standard Deviation).

		Mean	SD	Minimum	Maximum	Statistic	*p*
Attention						−1.837	0.067
	Men	25.27	6.69	10	39		
	Women	27.06	6.53	12	40		
Clarity						1.645	0.144
	Men	24.14	6.87	13	40		
	Women	22.71	6.48	10	40		
Repair						1.010	0.314
	Men	24.78	6.51	9	40		
	Women	23.83	6.31	12	40		
Attention						0.035	0.966
	Dentistry	26.69	6.52	13	40		
	Medicine	26.51	6.62	10	40		
	Pharmacy	26.79	6.84	14	40		
Clarity						1.304	0.273
	Dentistry	22.31	5.88	11	40		
	Medicine	23.76	7.12	12	40		
	Pharmacy	23.40	7.07	10	39		
Repair						0.282	0.754
	Dentistry	23.72	6.24	12	40		
	Medicine	24.38	6.47	9	39		
	Pharmacy	24.21	6.49	13	40		

**Table 2:** Summary of descriptive statistics and statistical comparisons related to the scores for each personality trait (NEO-FFI), by gender and degree program. (SD: Standard Deviation).

		Mean	SD	Minimum	Maximum	Statistic	*p*
Neuroticism						2.463	0.014
	Men	37.08	9.01	16	56		
	Women	40.20	8.39	18	57		
Extraversion						−0.625	0.533
	Men	41.34	9.05	15	60		
	Women	42.04	7.07	13	58		
Openness to experience						0.477	0.634
	Men	42.34	7.33	25	56		
	Women	41.86	6.62	28	59		
Agreeableness						0.031	0.976
	Men	42.05	4.92	26	52		
	Women	42.03	5.83	20	54		
Conscientiousness						−0.020	0.984
	Men	43.08	7.88	23	58		
	Women	43.11	7.07	20	58		
Neuroticism						1.000	0.369
	Dentistry	39.62	8.15	20	55		
	Medicine	38.55	9.11	16	57		
	Pharmacy	40.61	8.79	18	56		
Extraversion						1.955	0.144
	Dentistry	42.90	5.99	25	55		
	Medicine	40.93	9.17	16	60		
	Pharmacy	41.23	7.62	23	54		
Openness to experience						10.481	<0.001
	Dentistry	40.44	6.02	25	56		
	Medicine	44.60	6.90	27	59		
	Pharmacy	41.12	6.99	27	56		
Agreeableness						1.094	0.337
	Dentistry	41.58	5.63	20	54		
	Medicine	42.75	5.56	26	53		
	Pharmacy	41.86	5.70	29	54		
Conscientiousness						3.986	0.020
	Dentistry	44.08	6.66	29	57		
	Medicine	43.31	7.47	20	58		
	Pharmacy	40.82	7.65	25	58		

**Table 3:** Correlation coefficients between the components of emotional intelligence and the personality traits.

		Attention	Clarity	Repair
**Neuroticism**	r	0.272	−0.330	−0.439
	*p*	<0.001	<0.001	<0.001
	Effect size	Small	Medium	Medium
**Extraversion**	r	0.134	0.192	0.332
	*p*	0.032	0.002	<0.001
	Effect size	Small	Small	Medium
**Openness**	r	0.386	0.180	0.143
	*p*	<0.001	0.004	0.022
	Effect size	Medium	Small	Small
**Agreeableness**	r	0.134	0.156	0.337
	*p*	0.032	0.012	<0.001
	Effect size	Small	Small	Medium
**Conscientiousness**	r	0.048	0.135	0.172
	*p*	0.442	0.030	0.006
	Effect size	-	Small	Small

## Data Availability

No new data were created or analyzed in this study. Data sharing is not applicable to this article.
